# Endoscopic Screening for Second Primary Tumors of the Esophagus Among Head and Neck Cancer Patients

**DOI:** 10.3389/fonc.2022.906125

**Published:** 2022-06-07

**Authors:** Chen-Shuan Chung, Li-Jen Liao, Chia-Yun Wu, Wu-Chia Lo, Chen-Hsi Hsieh, Tzong-His Lee, Chao-Yu Liu, Deng-Yu Kuo, Pei-Wei Shueng

**Affiliations:** ^1^ Division of Gastroenterology and Hepatology, Department of Internal Medicine, Far Eastern Memorial Hospital, New Taipei City, Taiwan; ^2^ College of Medicine, Fu Jen Catholic University, New Taipei City, Taiwan; ^3^ Head and Neck Cancer Surveillance & Research Group, Far Eastern Memorial Hospital, New Taipei City, Taiwan; ^4^ Otolaryngology Head and Neck Surgery, Far Eastern Memorial Hospital, New Taipei City, Taiwan; ^5^ Department of Electrical Engineering, Yuan Ze University, Taoyuan, Taiwan; ^6^ Division of Medical Oncology, Department of Internal Medicine, Far Eastern Memorial Hospital, New Taipei City, Taiwan; ^7^ Graduate Institute of Medicine, Yuan Ze University, Taoyuan, Taiwan; ^8^ Institute of Traditional Medicine, School of Medicine, National Yang Ming Chiao Tung University, Taipei, Taiwan; ^9^ Division of Radiation Oncology, Department of Radiology, Far Eastern Memorial Hospital, New Taipei City, Taiwan; ^10^ School of Medicine, College of Medicine, National Yang Ming Chiao Tung University, Taipei, Taiwan; ^11^ Division of Thoracic Surgery, Department of Surgery, Far Eastern Memorial Hospital, New Taipei City, Taiwan; ^12^ Medical Device Innovation and Translation Center, National Yang Ming Chiao Tung University, Taipei, Taiwan

**Keywords:** head neck cancer, esophageal cancer, second primary tumor, cancer screening, image-enhanced endoscopy, narrow-band imaging, Lugol’s chromoendoscopy

## Abstract

Malignancies of the head and neck (HN) region and esophagus are among the most common cancers worldwide. Due to exposure to common carcinogens and the theory of field cancerization, HN cancer patients have a high risk of developing second primary tumors (SPTs). In our review of 28 studies with 51,454 HN cancer patients, the prevalence of SPTs was 12%. The HN area is the most common site of SPTs, followed by the lungs and esophagus, and 13% of HN cancer patients have been reported to have esophageal high-grade dysplasia or invasive carcinoma. The prognosis of HN cancer patients with concomitant esophageal SPTs is poor, and therefore identifying esophageal SPTs as early as possible is of paramount importance for risk stratification and to guide the treatment strategy. Image-enhanced endoscopy, especially using narrow-band imaging endoscopy and Lugol’s chromoendoscopy, has been shown to improve the diagnostic performance in detecting esophageal neoplasms at an early stage. Moreover, the early detection and minimally invasive endoscopic treatment of early esophageal neoplasm has been shown to improve the prognosis. Well-designed prospective studies are warranted to establish appropriate treatment and surveillance programs for HN cancer patients with esophageal SPTs.

## Introduction

Malignancies of the head and neck (HN) region and esophagus are among the most common cancers worldwide (1). In parallel with the advances in diagnostic modalities for cancer screening and surveillance, an increasing number of second primary tumors (SPTs) are being detected. SPTs may develop into any kind of malignancy, including malignancy of multicentric origins in the HN region, lungs and esophagus, particularly in HN cancer patients (2–5). This cancerization field known as the upper aerodigestive tract (UADT) is exposed to common carcinogens, particularly cigarette smoke, alcohol, and betel quid. The occurrence of SPTs in the UADT, either synchronously or metachronously, and single or multiple, in HN cancer patients is associated with worse survival despite appropriate management of the primary index HN tumor (2, 3, 6, 7). Of these SPTs, esophageal cancer is associated with a worse prognosis than other sites of the UADT (2, 3). Moreover, esophageal SPTs are easily overlooked as many are diagnosed at asymptomatic early stages (8–12). Therefore, the early identification of esophageal neoplasms and treatment of the primary index cancer and esophageal SPTs is of paramount importance to improve the overall outcomes of HN cancer patients. In this review, we describe the association between HN and esophageal cancers, and propose a screening strategy for esophageal SPTs among HN cancer patients.

## Disease Burden of HN Cancer and Esophageal Cancer

Head and neck cancers are the sixth and seventh most common cancers in Taiwan and worldwide, respectively (1, 13). Globally, HN cancer was the fifth most common cancer in men and the 12th most common cancer in women, accounting for an estimated 8,170 and 888,000 new cases in Taiwan and worldwide, respectively, in 2018 (1, 13). The incidence is higher in males, especially middle-aged males, with a male-to-female incidence ratio of 3:1, and most (about 70%) new cases occur in low- and middle-income countries (1). Regarding mortality from HN cancer, there were an estimated 3,027 and 453,000 deaths in Taiwan (the fifth leading cause of cancer deaths) and worldwide, respectively, in 2018 (1, 13). A Canadian study examined the 25-year survival outcomes of 1,657 patients, and reported 2, 5, 15 and 25-year HN cancer-specific survival rates of 74%, 63%, 53% and 49%, respectively (14). In addition, an Italian study of 801 cases reported a 5-year overall survival for HN cancer of 62%, including 55% for cancer of the oral cavity, 53% for the oropharynx, 41% for the hypopharynx, and 71% for the larynx (15). In Taiwan, the 5-year overall survival for HN cancer during the past decade ranged from 40~60%, and the standardized death growth rate in men was 7.7% (13).

Esophageal cancer is the eighth most common cancer (sixth in Taiwanese males) and the sixth most common cause of cancer deaths (ninth in Taiwan) worldwide (1, 13). Malignancy of the esophagus has two main histological subtypes, namely esophageal squamous cell carcinoma (ESCC), and esophageal adenocarcinoma (EAC). ESCC accounts for the majority (93.13% in Taiwan, 87% globally) of all esophageal cancer cases (1, 13). In 2012, there were an estimated 398,000 and 52,000 new cases of ESCC and EAC, respectively, worldwide (1). In Taiwan, 2,436 and 84 new cases of ESCC and EAC were reported in 2018 (13). The male-to-female incidence ratio is 2.7:1 for ESCC and 4.4:1 for EAC (1). Similar to HN cancer, about half (52.71%) of esophageal cancers develop in patients aged between 40~60 years in Taiwan (13). The overall prognosis of esophageal cancer is poor because most cases are diagnosed at a late stage with obstructive symptoms. Only 15.93% of esophageal cancer patients are diagnosed at stage 0/I, compared to 69.83% at stage III/IV in Taiwan (13). The overall 5-year survival rate for esophageal cancer is less than 10~20%, and lower than 5% in low- and middle-income countries (1, 13, 16). In Taiwan, the standardized death growth rate of esophageal cancer during the past decade was 15.5% (13).

The incidence rates of both HN and esophageal cancers are increasing and the prognosis is unsatisfactory, especially for esophageal cancer. Most cases occur in middle-aged males with a great impact on cancer-related morbidity and mortality. Consequently, early detection through screening programs for patients at high risk is crucial to improve their prognosis.

## Association Between HN and Esophageal Cancers

### Common Risk Factors and the Epidemiology for HN Cancer and Esophageal SPTs

The risk factors for HN cancer include male sex, infectious agents [*human papillomaviruses (HPV)*, *Epstein–Barr virus*], exposure to carcinogens (tobacco or marijuana use, alcohol consumption, betel quid chewing), poor oral hygiene, history of esophageal cancer, drinking hot beverages such as maté, occupational exposure (metal smelting and textile production), and consumption of preserved foods with high nitrosamine content (1, 13, 17–19). In addition, genetic factors have also been associated with the development of HN cancer. Among non-*HPV*-related HN cancers, *TP53* and cyclin-dependent kinase inhibitor 2A (*CDKN2A*) are the most affected genes, while the genetic changes in *HPV*-related tumors are in the phosphoinositide 3-kinase (PI3K) pathway, particularly involving activating mutations and amplifications of the *PIK3CA* oncogene (1, 6). Alcohol-metabolizing enzyme gene polymorphisms have also been associated with a higher risk of HN cancer (19, 20). For ESCC, the risk factors are older age, male sex, low body mass index, lower socioeconomic status, exposure to carcinogens (alcohol consumption, cigarette smoking, and betel quid chewing), low fruit/vegetable consumption, high meat/high temperature beverage intake, family members with esophageal cancer, history of HN cancer, poor oral hygiene, genetic polymorphism of alcohol-dehydrogenase-1B (ADH1B) and aldehyde dehydrogenase-2 (ALDH2), and motor disorders of the esophagus (e.g., achalasia) (7, 19, 21). For EAC, the most important risk factors are obesity, gastroesophageal reflux disease and Barrett’s esophagus (7). Mutations of tumor suppressor genes, multiple allelic losses, hypermethylation of promoter genes, genetic overexpression, and changes in miRNA expression profile have also been reported in both EAC and ESCC (7).

**Figure 1:**
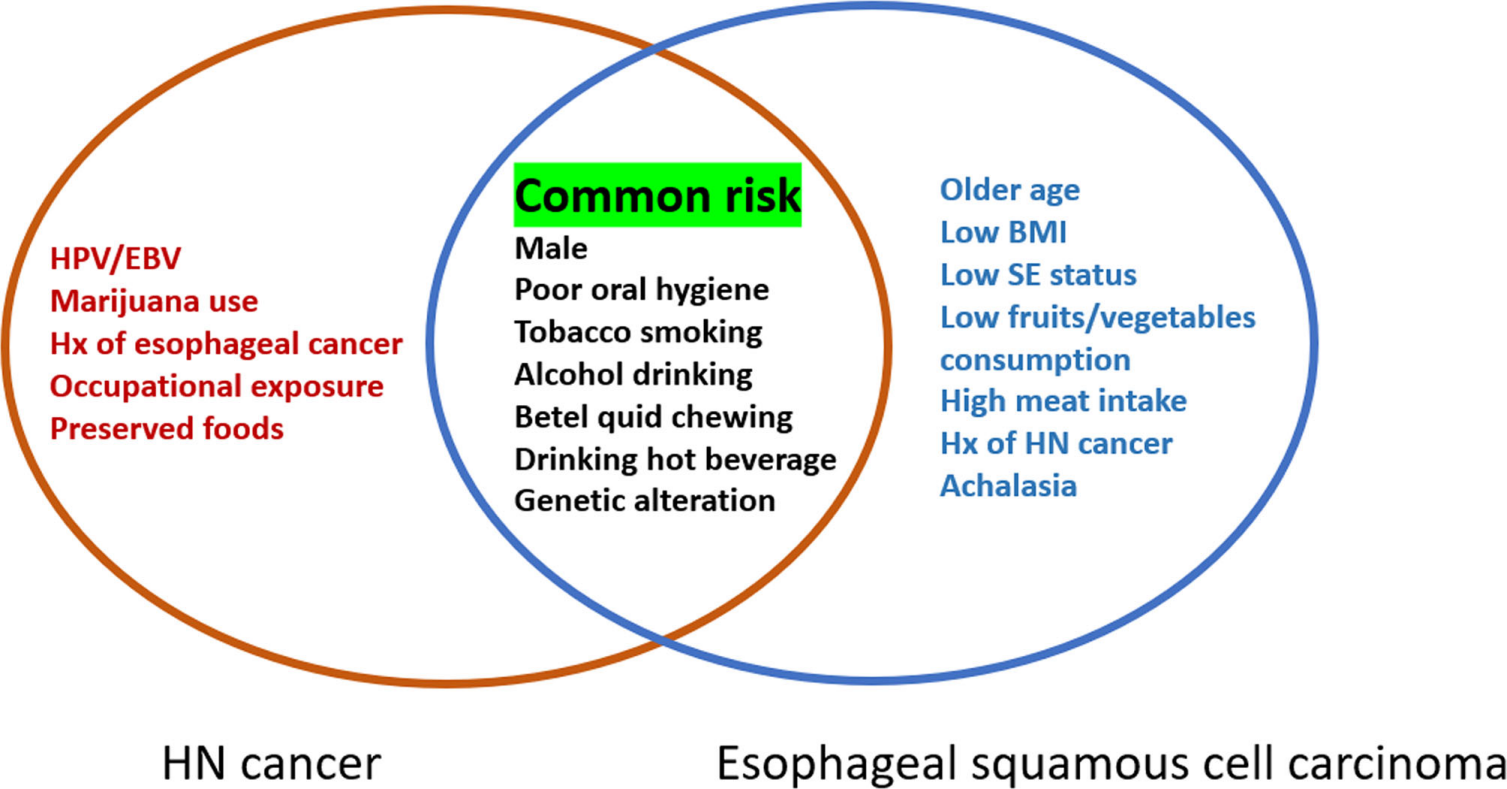
Risk factors for head and neck cancer and esophageal squamous cell carcinoma. HPV, human papillomavirus; EBV, Epstein-Barr virus; Hx, history; BMI, body mass index; SE, socioeconomic.

**Figure 2:**
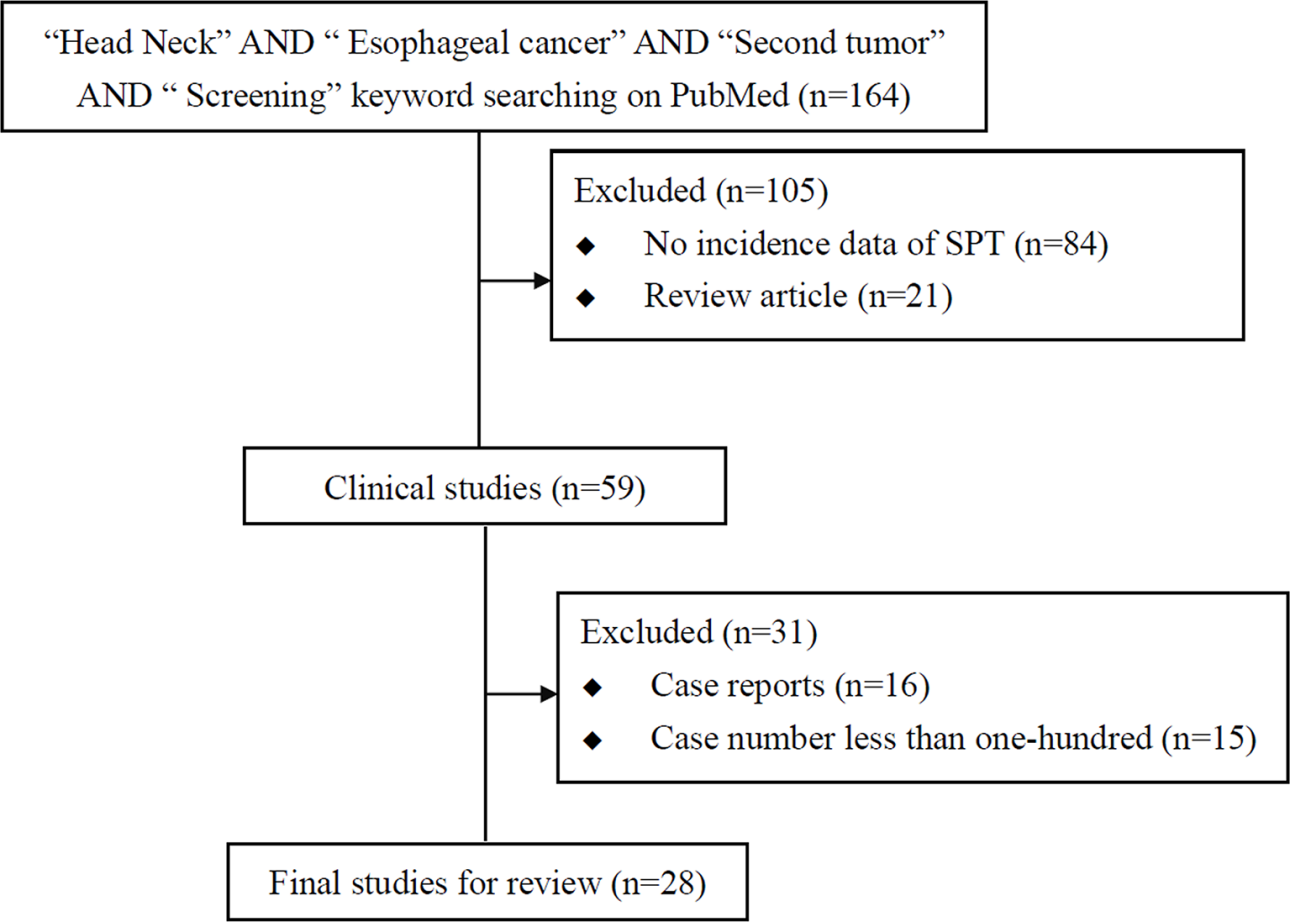
Flowchart of literature review of studies on screening esophageal second primary tumor (SPT) in head and neck cancer patients.

**Figure 3:**
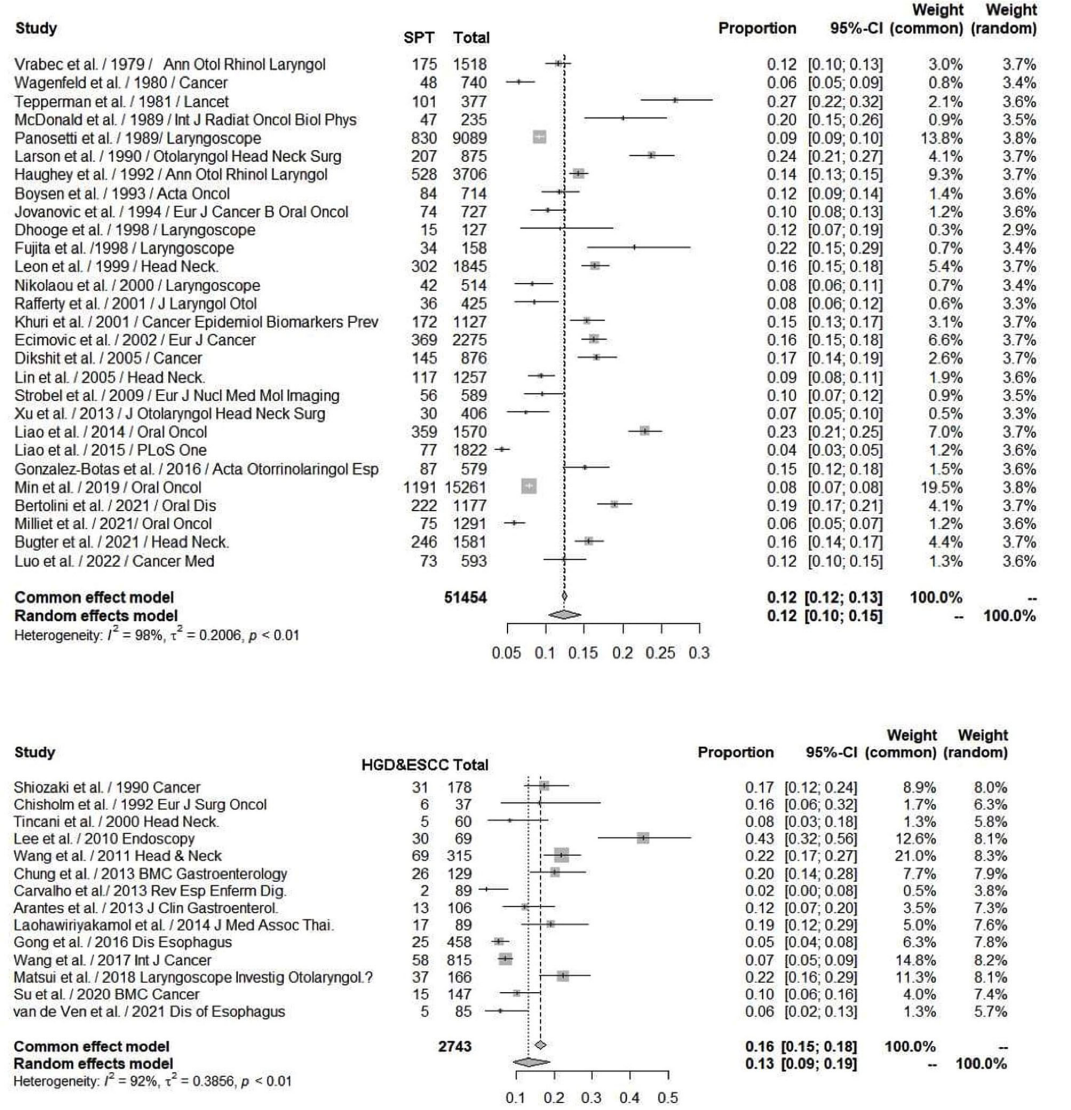
*Upper*: Forest plots showing the reported proportion of SPTs among head and neck cancers with a random effect models due to significant heterogeneity, the overall SPT rate was 12% (95% CI, 10-15%). *Lower*: Forest plots showing a reported 13% incidence rate of HGD and ESCC (95% CI, 9-19%) by image-enhanced endoscopy screening among head and neck cancer patients. ESCC, esophageal squamous cell carcinoma; HGD, high-grade dysplasia; SPT, second primary tumor.

There are many common risk factors for the development of HN cancer and ESCC. The squamous epithelium of both the HN region and esophagus are exposed to common environmental factors, particularly carcinogens. Consequently, with underlying genetic alterations such as polymorphisms in alcohol-metabolizing enzyme genes, those with accumulating exposure to carcinogens may develop both HN cancer and ESCC (**Figure 1**). Several epidemiology studies have demonstrated an increased risk of synchronous and metachronous SPTs among HN cancer patients. We used keywords including “head and neck” AND “ esophageal cancer” AND “ second tumor” AND “screening” for literature review on PubMed. Exclusion criteria were as followings: studies without data upon incidence of esophageal SPTs, review article, case reports and number of HN cancer patients less than one-hundred (**Figure 2**). In our review of 28 studies with 51,454 HN cancer patients, the estimated prevalence of SPTs was 12% (95% CI, 10-15% with a random effects model). The index primary cancer, sites of SPT, and screening modalities in these 51,454 HN cancer patients are shown in **Table 1** and **Figure 3** (3, 8, 11, 12, 17, 22–45, 47–51). One 10-year follow-up study of 6,258 HN cancer patients reported that 21.8% presented with SPTs, with the highest excess absolute risk (EAR) for SPTs of the lungs, followed by those located at the HN region and esophagus (52). Similar results were reported in a population-based cohort study of 64,673 HN cancer patients in the National Cancer Institute Surveillance, Epidemiology, and End Results (SEER) registry between 1979 and 2008, in which the standardized incidence ratio (SIR) of synchronous SPTs was 5.0, with the highest excess risk of a second cancer at the HN region (SIR, 41.4), followed by the esophagus (SIR, 21.8), and lungs (SIR, 7.4) (53). In addition, a meta-analysis reported an SIR for metachronous SPTs, which were defined as occurring six months after the primary index tumor, of 2.04 (95% CI, 1.61~2.59) (9). The highest risk for metachronous SPTs located at the HN region was for the oropharynx (SIR, 17.82; 95% CI, 6.79–46.77), followed by the hypopharynx (SIR, 9.17; 95% CI, 3.51–23.98) and larynx (SIR, 4.12; 95% CI, 2.87–5.90), while the highest risk for SPTs located outside the HN area was for the esophagus (SIR, 4.64; 95% CI, 3.12–6.89), followed by the salivary glands (SIR, 8.30; 95% CI, 2.37–29.09) and thyroid (SIR, 1.47; 95% CI, 1.22–1.76) (9). In a study that defined a metachronous SPT as occurring 2 months after the primary HN cancer, an increased risk for metachronous SPTs of the lungs (SIR, 4.32; 95% CI 2.15-8.68) was also noted (9). Another systematic review of 456,130 HN cancer patients from 61 articles with a minimum follow-up of 22 months reported a mean incidence of SPTs of 13.2% (95% CI, 11.56-14.84), including 5.3% for synchronous SPTs (95% CI, 4.24-6.36) and 9.4% for metachronous SPTs (95% CI, 7.9-10.9) (54). In addition, the most common site of SPTs was the HN area, followed by the lungs and esophagus, which is similar to other studies (54). Metachronous SPTs are more prevalent than synchronous SPTs, and therefore, surveillance programs including investigations for SPTs are of paramount importance to improve the long-term care of HN cancer patients (17, 55).

**Table 1:** Prevalence of SPT in HN cancer patients.

Author/Reference no.Year	No (%) of SPT/All/Index HN cancer	Esophagus,no (%)	Lung,no (%)	HN region,no (%)	Others,no (%)
**Vrabec** (22) **1979**	175 (11.5)/1,518/Oral cavity, pharynx or larynx	25 (14.3)	49 (28.0)	49 (28.0)	52 (29.7)
**Wagenfeld** (23) **1980**	48 (6.5)/740/Glottis	3 (6.3)	25 (52.1)	20 (41.7)	0 (0)
**Tepperman** (24) **1981**	101 (26.8)/377/Oral cavity	10 (9.9)	24 (23.8)	48 (47.5)	19 (18.8)
**McDonald** (25)	47 (20)/235/Larynx	0 (0)	22 (46.8)	9 (19.1)	16 (34.0)
**Panosetti** (17) **1989**	830 (9.1)/9,089/Oral cavity, pharynx, larynx	103 (12.4)	89 (10.7)	398 (47.9)	240 (28.9)
**Larson** (26) **1990**	207 (23.7)/875/Oral cavity, pharynx, larynx	13 (6.3)	54 (26.1)	129 (62.3)	11 (5.3)
**Haughey** (27) **1992**	528 (14.2)/3,706/Oral cavity, pharynx and larynx	17 (3.2)	106 (20.1)	246 (46.6)	159 (30.1)
**Boysen** (28) **1993**	84 (11.8)/714/Oral cavity, pharynx, larynx	10 (11.9)	19 (22.6)	29 (34.5)	26 (31.0)
**Jovanovic** (29) **1994**	74 (10.2)/727/lip and oral cavity	8 (10.8)	19 (25.7)	47 (63.5)	0 (0)
**Dhooge** (30) **1998**	15 (11.8)/127/Oral cavity, pharynx, larynx, cervical esophagus	4 (26.7)	6 (40.0)	5 (33.3)	0 (0)
**Fujita** (31) **1998**	34 (21.5)/158/Larynx	2 (5.9)	14 (41.2)	8 (23.5)	10 (29.4)
**León** (32) **1999**	302 (16.4)/1,845/Oral cavity, pharynx, and larynx	27 (8.9)	100 (33.1)	122 (40.4)	53 (17.5)
**Nikolaou** (33) **2000**	42 (8.2)/514/Larynx	12 (28.6)	13 (31.0)	5 (11.9)	12 (28.6)
**Rafferty** (34) **2001**	36 (8.5)/425/Oral cavity, pharynx, and larynx	3 (8.3)	6 (16.7)	27 (75.0)	0 (0)
**Khuri** (35) **2001**	172 (15.3)/1,127/Oral cavity, pharynx, larynx	6 (3.5)	57 (33.1)	50 (29.1)	59 (34.3)
**Ećimović** (36) **2002**	369 (16.2)/2,275/Larynx	15 (4.1)	155 (42.0)	81 (21.9)	118 (32.0)
**Dikshit** (37) **2005**	145 (16.6)/876/Larynx and hypopharynx	15 (10.3)	55 (37.9)	52 (35.9)	23 (15.9)
**Lin** (38) **2005**	117 (9.3)/1,257/Oral cavity and larynx	7 (5.9)	48 (41.0)	40 (34.2)	22 (18.8)
**Strobel** (39) **2009**	56 (9.5)/589/Oral cavity, pharynx, and larynx	5 (8.9)	26 (46.4)	15 (32.6)	10 (17.9)
**Xu** (40) **2013**	30 (7.4)/406/oropharynx	1 (3.3)	7 (23.3)	19 (63.3)	3 (10.0)
**Liao** (41) **2014**	359 (22.9)/1,570/Oral cavity	14 (3.9)	25 (7.0)	281 (78.3)	39 (10.9)
**Liao** (42) **2015**	77 (4.2)/1,822/Oral cavity	4 (5.2)	0 (0)	66 (85.7)	7 (9.1)
**González-Botas** (43) **2016**	87 (15.0)/579/Oral cavity, pharynx, and larynx	5 (5.7)	32 (36.8)	33 (37.9)	17 (19.5)
**Min** (44) **2019**	1,191 (7.8)/15,261/Oral cavity	92 (7.7)	250 (21.0)	168 (14.1)	681 (57.2)
**Bertolini** (45) **2021**	222 (18.9)/1,177/Oral cavity, pharynx, and larynx	9 (4.1)	67 (30.2)	70 (31.5)	76 (34.2)
**Milliet** (46) **2021**	75 (5.8)/1,291/Oropharynx	7 (9.3)	13 (17.3)	50 (66.7)	5 (6.7)
**Bugter** (47) **2021**	246 (15.6)/1,581/Oral cavity, pharynx, and larynx	23 (9.3)	82 (33.3)	141 (57.3)	0 (0)
**Luo** (48) **2022**	73 (12.3)/593/Hypopharynx	23 (31.5)	13 (17.8)	14 (19.2)	23 (31.5)
**All reviewed studies**	**5,742 (11.2)/51,454/HN region**	**463 (8.1)**	**1,376 (23.9)**	**2,222 (38.7)**	**1,681 (29.3)**

HN, head and neck; SPT, second primary tumor. The bold values were the summary data of enrolled studies.

### Different Risk for Esophageal SPTs According to the Primary Site of HN Cancer

The risk factors for SPTs are different depending on the primary site of the index HN cancer. One study of 75,087 HN cancer patients in the SEER database reported the highest risk for SPTs for primary hypopharyngeal cancer (SIR, 3.5; EAR, 307.1 per 10,000 person-years) and the lowest for laryngeal cancer (SIR, 1.9; EAR, 147.8 per 10,000 person-years) (56). Nasopharyngeal cancer (NPC) arises from a unique site with a large number of resident leukocytes, predominantly T-cells, together with other stromal cells. Therefore, the pathophysiology and tumor phenotype of NPC is quite different from other HN cancers, and the reported association between NPC and ESCC is lower than for other primary sites in the HN region. One large retrospective study of a cohort of 1,549 NPC patients following radiotherapy in Taiwan reported increased risks of developing SPTs in the HN region (SIR, 16.5; 95% CI, 10.0~26.8), stomach (SIR, 5.5; 95% CI, 2.2~11.4) and leukemia (SIR, 9; 95% CI, 1.9~26.3) (57). In a multicenter study of 8,947 NPC patients, 167 (1.9%) patients developed SPTs with increased risks of tongue cancer, non-Hodgkin’s lymphoma, brain cancer, myeloid leukemia and non-melanoma skin cancer (58). Interestingly, the risk of developing SPTs has been shown to vary between different histological subtypes among NPC patients. A cross-sectional study of 1,175 NPC patients reported that SPTs, and especially those located in the HN region and UADT, were more prevalent in keratinizing NPC compared to non-keratinizing NPC (59). Another multicenter study of 3,166 NPC patients also reported significantly higher risks of cancer in the oral cavity, sarcoma, oropharynx, paranasal sinus, salivary gland, thyroid, skin and lungs (60).

Of note, a significantly lower risk of SPTs has been demonstrated among patients with oropharyngeal SCC in the *HPV* infection era (annual percentage change in EAR, -4.6%; p = 0.03), and that routine panendoscopy examinations are not even recommended in some studies (56, 61). A Canadian retrospective study of 406 oropharyngeal cancer patients reported a significantly lower incidence rate of SPTs in those who were p16-positive, which is indicative of *HPV*-related oropharyngeal cancer patients (0.7 per 100 patient-years vs. 8.5 in p16-negative patients, p < 0.0001) (40). In addition, the yield rate of field cancerization work-up (2.8% vs. 10.2%, p = 0.02) was lower in the *HPV*-positive than in the *HPV*-negative oropharyngeal cancer patients (40). Moreover, multivariate analysis from a multicenter study of 1,291 HN cancer patients showed that p16-negative tumor status (p = 0.003), tobacco/alcohol consumption (p = 0.005), and soft palate tumor site (p = 0.009) were significantly associated with a higher risk of metachronous SPTs (46). Furthermore, a higher proportion of metachronous SPTs arising outside the UADT was found in *HPV*-positive than in *HPV*-negative patients (46).

### Second Primary Tumors of HN Region in Primary Esophageal Cancer Patients

Second primary neoplasms occur mutually in patients with UADT cancers. Patients with primary ESCC are also at risk of SPTs in the HN region. Analysis of data from a mean follow-up period of 76 months in a study of 285 ESCC patients showed 5-year cumulative occurrence rates of metachronous SPTs of the esophagus, HN region and stomach of 14.0%, 2.8% and 4.1%, respectively (62). Another study of 439 superficial esophageal cancer patients reported that 53 metachronous HN cancers developed in 40 (9.1%) patients after a median follow-up period of 46 months, and the cumulative incidence rates of metachronous HN cancers at 3, 5, and 7 years were 5.3%, 9.7%, and 17.2%, respectively (63). A systematic review of 6,483 ESCC patients from 12 studies in Japan revealed a pooled prevalence of HN SPTs of 6.7% (95% CI, 4.9~8.4%), including 48.2% synchronous and 51.8% metachronous SPTs, 85.3% at an early stage, and 60.3% located in the hypopharynx (18).

### Prognosis of HN Cancer Patients With Esophageal SPTs

Esophageal SPTs not only occur synchronously or metachronously, but also have a negative impact on the prognosis of HN cancer patients (64). The 15-year survival rate of HN cancer patients with SPTs is lower than in those without SPTs (22% vs. 54%), and the prognosis is especially poor with a 5-year survival rate of only 6% in those with esophageal SPTs (vs. 25% in those with all SPTs) (2, 3, 26). Another study also demonstrated lower 5-year (68% vs. 76%) and 10-year (26% vs. 57%) overall survival rates in laryngeal cancer patients who developed SPTs (p = 0.003) (31). A nationwide analysis of 93,891 HN cancer patients from the Taiwan Cancer Registry reported that 9,996 (10.6%) patients presented with SPTs, and that those with SPTs had a significantly lower survival rate (univariate analysis: HR, 2.59; 95% CI, 2.53-2.65; multivariate analysis: HR, 2.34; 95% CI, 2.28-2.40) (65).

To summarize, the risk and distribution of SPTs differ significantly according to the subsite of the index primary HN cancer, with a lower risk in laryngeal and *HPV*-positive oropharyngeal cancer patients. About 11.2% of HN cancer patients develop either synchronous or metachronous SPTs at the HN region (38.7%), lung and bronchus (23.9%), and esophagus (8.1%) (**Table 1**). The occurrence of ESCC is especially associated with a poor prognosis, and thus identifying esophageal SPTs is crucial in screening and surveillance programs for HN cancer patients.

## Image-Enhanced Endoscopic Screening and Risk Factors for Esophageal SPTs in HN Cancer Patients

**Figure 4:**
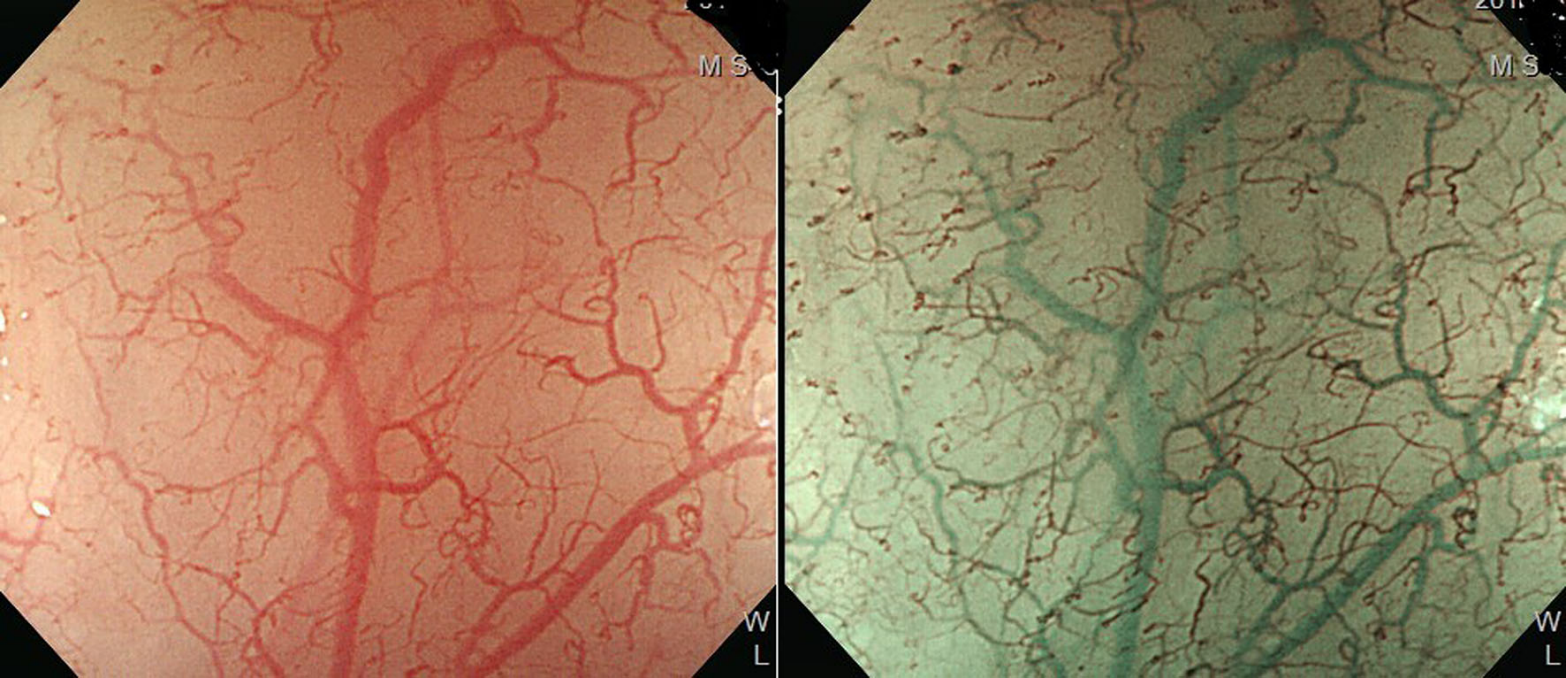
Improved visualization of microvascular structure under narrow-band imaging endoscopy (*Left*: conventional white-light imaging. *Right*: narrow-band imaging.).

**Figure 5:**
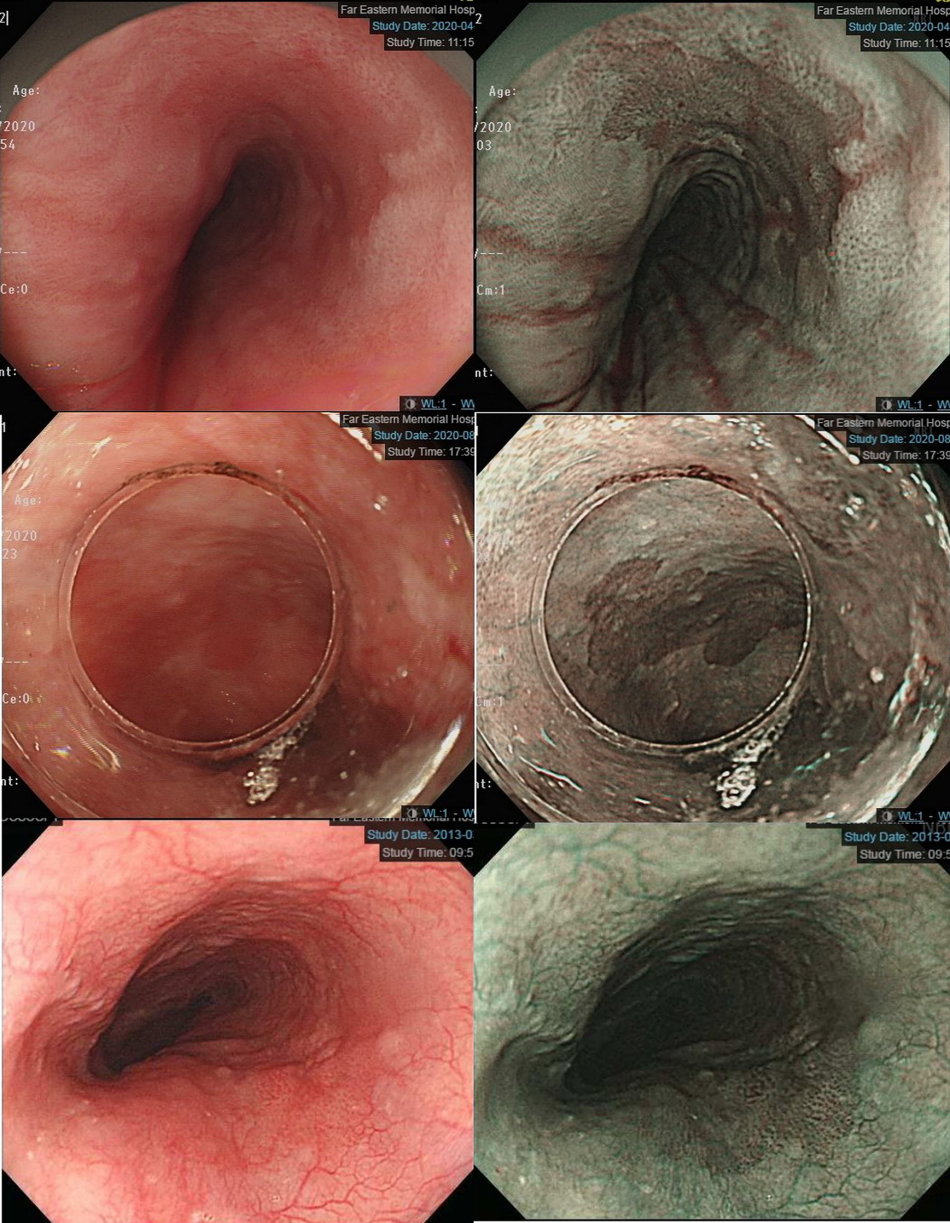
*Left panels*: Early esophageal neoplasm with barely visible flat morphology under conventional white-light endoscopy. *Right panels*: Dark brownish color compared with the greenish color of healthy mucosa under narrow-band imaging endoscopy.

**Figure 6:**
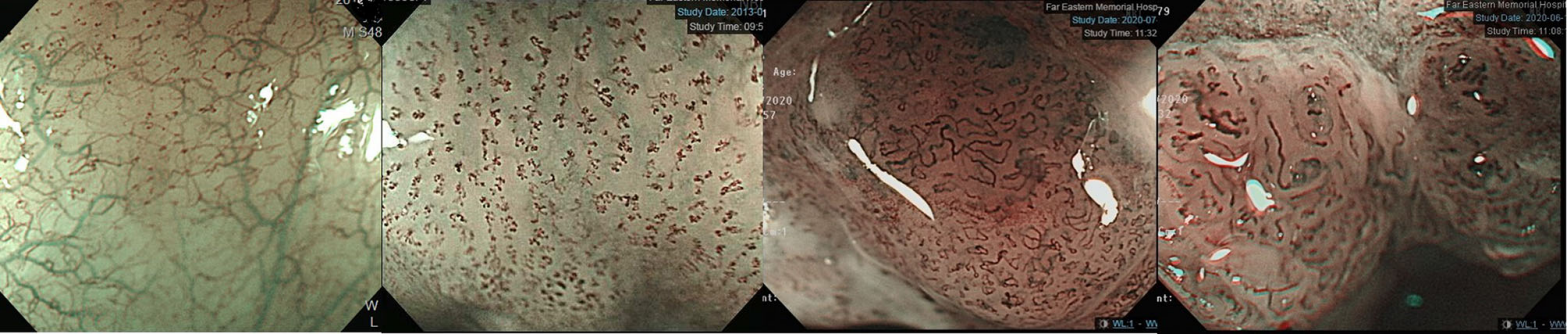
JES classification of microvessel morphology of IPCL. *From left to right*: **JES type A-** Normal IPCL without irregularity. **JES type B1-** Abnormal microvessels with severe irregularity, meandering caliber or highly dilated proliferative abnormal vessels with a loop-like formation. **JES type B2-** Abnormal microvessels with severe irregularity, meandering calibers or highly dilated proliferative abnormal vessels without a loop-like formation. **JES type B3-** Highly dilated microvessels with three times as many calibers than usual type B2 vessels. IPCL, intraepithelial papillary capillary loop; JES, Japanese Esophageal Society.

**Figure 7:**
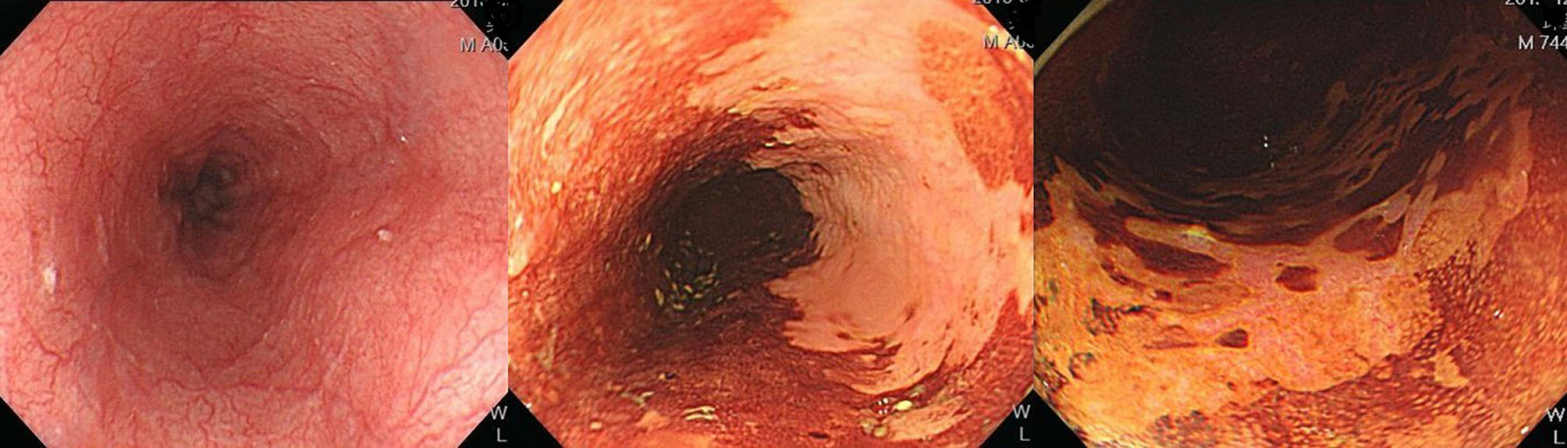
Esophageal high-grade dysplastic lesion. *Left*: Normal appearance upon white-light endoscopy. *Middle*: Lugol-voiding unstained mucosa. *Right*: The color of Lugol-unstained mucosa turns pink in a few minutes.

Esophagogastroduodenoscopy is the most reliable diagnostic tool for esophageal neoplasms, especially using an image-enhanced endoscopy (IEE) system, which is composed of optical- and dye-based technology (49, 66, 67). Among several IEE techniques, narrow-band imaging (NBI) and chromoendoscopy with Lugol’s solution are widely used for screening ESCC (49, 66–68). By using narrow-bandwidth filters to remove red light and narrow wavelengths of green (540 nm) and blue (415 nm) light, NBI can improve visualization of hemoglobin-rich vascular microstructures (**Figure 4**) (49). Because the color of gastrointestinal mucosa is primarily determined by hemoglobin, and neovascularization occurs in neoplastic squamous epithelium of the esophagus, the light emitted from NBI is absorbed by neoplastic mucosa more than healthy mucosa. Therefore, early neoplasms, which usually have a flat morphology, can be differentiated from normal mucosa by dark brownish discoloration compared with the greenish color of healthy mucosa under NBI (**Figure 5**). In addition, when combining a magnifying endoscope with an NBI system, the microvascular pattern of neoplastic squamous cell epithelium can be well delineated (**Figure 6**) (49, 67, 69). These microvessels seen under magnifying NBI, so-called intra-epithelial papillary capillary loops, can also predict tumor invasion depth with accuracy of 90.5% (69). Among dye-based IEE, iodine-containing Lugol’s solution is commonly used for ESCC screening. Normal glycogen-abundant squamous epithelium reacts with Lugol’s solution, while dysplastic mucosa with diminished or absent glycogen remains unstained (67, 68, 70). By spraying Lugol’s solution on esophageal mucosa, unstained areas are indicative of dysplastic or cancerous parts. Moreover, when unstained mucosa turns pink within a few minutes, high-grade dysplasia or squamous cell carcinoma can be diagnosed with a sensitivity of 91.9% and specificity of 94.0% (**Figure 7**) (68).

Both NBI and Lugol’s chromoendoscopy (LCE) are effective real-time screening endoscopic techniques for the early detection of esophageal neoplasms. A meta-analysis of 4,918 esophageal and HN cancer patients from 16 prospective and randomized trials showed that NBI and LCE had better diagnostic performance than conventional white-light imaging, with pooled sensitivity, specificity and area under the receiver operating characteristic curve of 87% (95% CI, 83~90%) and 88% (95% CI, 85~91%) versus 53% (95% CI, 48~59%), 99% (95% CI, 98~99%) and 95% (95% CI, 94~96%) versus 63% (95% CI, 61~66%), and 97% and 82% versus 66%, respectively (66). Given that most esophageal SPTs detected in HN cancer patients are at asymptomatic premalignant or early cancer stages, these lesions might be overlooked by white-light imaging or even advanced cross-sectional and radionuclide imaging modalities. In a study of 147 HN cancer patients, suspicious esophageal SPTs were identified by position emission tomography/computed tomography (PET/CT) in 8 (5.4%) and by NBI endoscopy in 35 (23.8%) patients (71). In addition, the diagnostic sensitivity of NBI endoscopy (100.0%) was superior to whole body PET/CT (33.3%) in detecting esophageal SPTs (71). In a review of 14 studies with 2,743 HN cancer patients, IEE screening identified esophageal high grade dysplasia or invasive carcinoma in 13% (95% CI, 9-19% with a random effects model) of the patients (**Table 2**, **Figure 3**) (8, 10–12, 70–79). Most of the esophageal SPTs were at an early stage without tumor-related obstructive symptoms. Therefore, if these esophageal SPTs had not been identified, the patients may have had a poor prognosis from esophageal cancer.

**Table 2:** Image-enhanced endoscopic screening of synchronous or metachronous esophageal neoplasm in HN cancer patients.

Author/Reference no.Year	Patient no./Study design/Endoscopy techniques	Incidence (excluding LGD) (%)/Lesions	Treatment
**Shiozaki** (72) **1990**	178 oral cavity, pharynx, larynx/Prospective/WLE, LCE	17.4/22 Dysplasia, 9 ESCC	CCRT, esophagectomy or laser
**Chisholm** (70) **1992**	37 oral cavity, pharynx, larynx/Prospective/WLE, LCE	16.2/6 ESCC	Not mentioned
**Tincani** (73) **2000**	60 oral cavity, pharynx, larynx/Prospective/WLE, LCE	8.3/5 ESCC	Esophagectomy
**Lee** (74) **2010**	69 oral cavity, pharynx, larynx/Prospective/WLE, NBI, LCE	30.4/5 LGD, 8 HGD, 22 ESCC	CCRT or esophagectomy for advanced cancers, ER for superficial neoplasm, or no treatment
**Wang** (11) **2011**	315 oral cavity, pharynx, larynx/Prospective/WLE, NBI, LCE	21.9/22 HGD, 47 ESCC	CCRT or esophagectomy for advanced cancers, ER for superficial neoplasm
**Chung** (8) **2013**	129 oral cavity, pharynx, larynx/Prospective/WLE, NBI, LCE	20.2/11 LGD, 14 HGD, 12 ESCC	Extended RT field or esophagectomy for advanced cancers, ER or radiofrequency ablation for superficial neoplasm
**Carvalho** (75) **2013**	89 oral cavity, pharynx, larynx/Prospective/WLE, LCE	2.2/2 HGD	ER
**Arantes** (76) **2013**	106 oral cavity, pharynx, larynx/Prospective/WLE, FICE	12.3/3 HGD, 10 ESCC	CCRT and ER
**Laohawiriyakamol** (77) **2014**	89 oral cavity, pharynx, larynx/Retrospective/WLE, LCE	12.4/6 Dysplasia, 11 ESCC	Not mentioned
**Gong** (78) **2016**	458 oral cavity, pharynx, larynx/Prospective/WLE, NBI, LCE	5.2/3 LGD, 15 HGD, 10 ESCC	CCRT or esophagectomy for advanced cancers, ER for superficial neoplasm, or no treatment
**Wang** (12) **2017**	815 oral cavity, pharynx, larynx/Prospective/WLE, NBI, LCE	7.1/66 LGD, 29 HGD, 29 ESCC	Not mentioned
**Matsui** (79) **2018**	166 oral cavity/retrospective/WLE, FICE, LCE	22.3/37 ESCC	CCRT or esophagectomy for advanced cancers, ER for superficial neoplasm
**Su** (71) **2020**	147 oral cavity, pharynx, larynx/Retrospective/WLE, NBI	10.2/5 HGD, 10 ESCC	Not mentioned
**van de Ven** (10) **2021**	85 oral cavity, pharynx, larynx/Prospective/WLE, NBI, LCE	5.9/3 LGD, 4 HGD, 1 ESCC	Extended RT field for advanced cancers, ER for superficial neoplasm

CCRT, concurrent chemoradiotherapy; ER, endoscopic resection; ESCC, esophageal squamous cell carcinoma; FICE, Fuji Intelligent Color Enhancement; HGD, high grade dysplasia; LCE, Lugol’s chromoendoscopy; LGD, low grade dysplasia; NBI, narrow band imaging; RT, radiotherapy; WLE, white-light endoscopy.

There are many common risk factors for HN and esophageal cancers. Among environmental factors, alcohol is one of the most important carcinogens for esophageal cancer (1, 19, 21). The results from a meta-analysis of 8 cohort and 11 case-control studies showed that alcohol drinking was associated with significantly increased risk of UADT SPTs (RR, 2.97; 95% CI, 1.96~4.50), and that every increase of 10 g/day in alcohol intake resulted in a significantly increased RR of 1.09 (95% CI, 1.04-1.14) for UADT SPTs in a dose-response relationship (80). Alcohol metabolizing enzyme genes are disease modifiers which are responsible for the increased risk of cancer after alcohol consumption (81). Ethanol is metabolized to acetaldehyde by alcohol dehydrogenase (ADH), then converted to acetate by acetaldehyde dehydrogenase (ALDH). The intermediate metabolized product, acetaldehyde is not only associated with unpleasant disulfiram-like reactions such as facial flushing, nausea, vomiting, tachycardia and hypotension, but also increased oxidant stress, inflammation and reactions with deoxynucleosides, leading to the formation of deoxyribonucleic acid adducts and subsequently cancerization (19, 81, 82). The results from a case-control study of 120 HN cancer and 138 ESCC patients in Taiwan demonstrated that the minor alleles of ADHB (rs1229984) and ALDH2 (rs671) were associated with an increased risk of UADT cancers (OR, 3.53 and 2.59; 95% CI, 2.14~5.80 and 1.79~3.75), and also that they potentiated the carcinogenic effects of alcohol (OR, 53.44 and 70.08; 95% CI, 25.21~113.29 and 33.65~145.95) (19). In addition, the haplotypes GAGC and CCAATG on chromosome 4 and 12, respectively, have been associated with a higher risk of HN and esophageal cancers (19). Another case-control study with age- and gender-matched 164 HN cancer patients showed that polymorphisms in ADH1B (OR, 2.09; 95% CI, 1.15~3.18; p < 0.05) and ALDH2 (OR, 5.19; 95% CI, 2.44~11.00; p < 0.001) increased the risk of developing multiple SPTs (20). Thus, HN cancer patients who are alcohol drinkers have a higher risk of esophageal SPT, particularly those carrying risk genetic polymorphisms of alcohol-metabolizing enzymes.

Primary sites of HN cancer are associated with different risk of developing esophageal SPTs. Compared with oral cavity and nasopharyngeal cancers, primary malignancy of the hypopharynx, *HPV*-negative oropharynx, and larynx are more likely to have esophageal SPTs (8, 11, 12, 50, 53, 54, 71). Other demographic data, including older age, comorbidities, lower body mass index, advanced stages of primary HN cancer and alcohol flushing syndrome have also been associated with a higher risk of esophageal SPTs (8, 12, 47). A systematic review identified 51 genes that were significantly associated with an increased risk of SPTs among HN cancer patients (83). In addition, the presence of multiple Lugol-voiding lesions, which are indicative of dysplastic or cancerous lesions in the esophagus, has also been reported to be a significant risk factor for developing both synchronous and metachronous SPTs (62, 84). A 13-year follow-up study of 682 patients with esophageal dysplasia reported that 23.7%, 50% and 73.9% of patients with low-grade, moderate, and high-grade dysplasia (HGD) developed invasive carcinoma (85). The molecular changes in Lugol-voiding mucosa precede the cancerization process, and the hotspot *p53* mutation has been identified in 20% and 40% of non-dysplastic and dysplastic Lugol-voiding mucosa (84). Therefore, when multiple Lugol-unstained areas are noted after LCE screening, a shorter interval of IEE surveillance for metachronous esophageal SPTs is mandatory.

**Figure 8:**
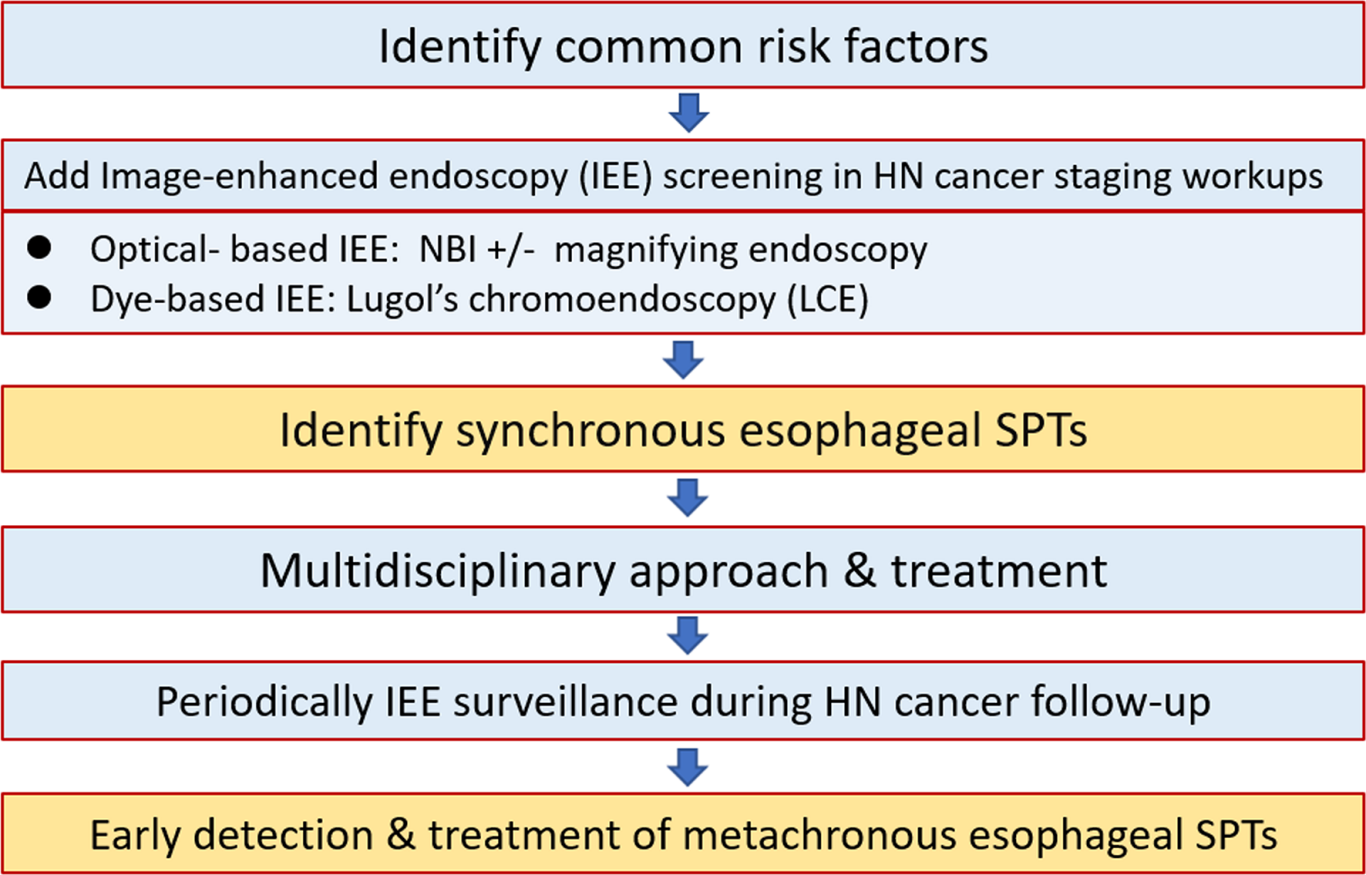
Approach algorithm for head and neck cancer patients at risk of esophageal second primary tumors. HN, head and heck; IEE, image-enhanced endoscopy; LCE, Lugol’s chromoendoscopy; NBI, narrow-band imaging; SPT, second primary tumor.

For HN cancer patients at risk of esophageal neoplasms, endoscopic screening and surveillance, especially using IEE techniques with NBI endoscopy and LCE, are crucial to identify esophageal SPTs. Before the development of obstructive symptoms from advanced esophageal neoplasms, the early detection of esophageal SPTs is one of the most important management strategies to improve the overall prognosis of HN cancer patients (**Figure 8**).

## Screening and Treatment Strategy of Esophageal SPTs for HN Cancer Patients

After screening for esophageal SPTs, HN cancer patients who are free from synchronous esophageal SPTs have the best outcomes (16). Thus, before starting treatment of newly diagnosed HN cancers, risk stratification and identification of synchronous esophageal SPTs could modify the oncological treatment plan (8). When considering ESCC treatment, surgical esophagectomy was the traditional curative therapeutic option. However, in the early 20th century, with advances in minimally invasive endoscopic resection techniques, early esophageal neoplasms could be managed by endoscopic submucosal dissection (ESD) and radiofrequency ablation (RFA) (86, 87). Due to the low risk of nodal or distant metastasis of superficial esophageal neoplasms, ESD can be considered as the first-line therapy for HGD or ESCC limited to the epithelium and lamina propria without lymphovascular invasion, while RFA can be considered for flat-type esophageal HGD or ESCC confined above the lamina propria (86–88). The overall curative resection and recurrence rates of esophageal neoplasms for ESD have been reported to be 78~100% and 0~2.6%, respectively, with complete remission and recurrence rates of 50~100% and 0~50% for RFA (86, 88). Five-year overall, disease-specific and metastasis-free survival rates above 90% have been reported after ESD for early esophageal neoplasms (86, 89, 90). Compared with surgical intervention, ESD (relative hazard, 0.89; 95% CI, 0.51~1.56; p = 0.68) has comparable long-term outcomes for early esophageal neoplasms, with a better quality of life and lower rate of adverse events (86, 90, 91). However, stricture complications are one of the most important concerns after ESD for large size neoplasms or those which involve more than 75% of the circumference (86, 90, 91). Most post-ESD strictures can be managed by endoscopic balloon dilation or prophylactic steroid therapy. As a result, identifying early esophageal SPTs in HN cancer patients could be a triage for screening and surveillance programs, and could also provide a chance for minimally invasive endoscopic resection with curative intent of early esophageal SPTs.

When considering the treatment strategy, the curability of both primary and secondary neoplasms must be carefully evaluated and discussed with a multidisciplinary approach. In HN cancer patients, prior treatment of the primary cancer often affects the treatment of esophageal SPTs. Trismus, malnutrition with cancer cachexia, performance status, the location of the esophageal SPT, and patient preference are important factors which should be taken into account. The treatment for esophageal SPTs, including endoscopic resection, concurrent chemoradiotherapy (CCRT), surgical intervention or no treatment, varies between studies due to the heterogeneous characteristics of HN cancer patients (**Table 2**). Cox proportional regression analysis of the SEER database which enrolled 3,038 HN cancer patients showed that those with SPTs of the HN region who underwent conservative surgery with radiation had the best 5-year overall survival rate (22.6%), those with lung SPTs who underwent radical surgery had the best 2-year overall survival rate (60.8%), and that there was no difference in the prognosis between treatment groups in those with esophageal SPTs (64). However, in a prospective study with long-term outcome analysis of 145 HN cancer patients, those with early esophageal SPTs who underwent aggressive treatment of both primary and secondary neoplasms had similar overall survival compared to HN cancer patients without esophageal SPTs (p = 0.47) (92). Definitive CCRT of esophageal cancer patients with synchronous HN SPTs can also safely be offered to improve overall survival, and those who receive CCRT have been shown to have better survival than those with radiotherapy alone (93).

Screening of esophageal SPTs by IEE should be performed in every newly diagnosed HN cancer patient, and regular IEE surveillance is also important to detect metachronous esophageal neoplasms. After identifying esophageal SPTs in HN cancer patients, management of neoplasms at the primary and secondary sites is quite complex and should be individualized according to the patient’s condition. It depends on the stage and survival of the primary and secondary tumors, prior treatments, expertise in endoscopic resection techniques and CCRT, as well as the patient’s performance and preference. Close cooperation between medical staff members including HN surgeon, gastroenterologist, endoscopist, oncologist and radio-oncologist are essential in a multidisciplinary approach.

## Summary

The development of synchronous or metachronous SPTs is more frequently being identified due to advances in diagnostic modalities, and it is an emerging issue in oncology medicine. SPTs are not uncommon among HN cancer patients, particularly those located in the HN region, lungs and esophagus. Patients with HN cancer and concomitant esophageal SPTs have the worst prognosis. Therefore, identifying esophageal SPTs in HN cancer patients is of paramount importance for risk stratification and to guide the treatment strategy. IEE, especially using NBI endoscopy and LCE, improves the diagnostic performance in detecting early esophageal neoplasms. Several studies have demonstrated a high diagnostic yield of IEE to identify esophageal SPTs at an early stage in HN cancer patients, particularly in patients at high risk, such those with primary sites of the hypopharynx and larynx, alcoholism with flushing syndrome, older age, and advanced stage primary HN cancer. In addition, with minimally invasive endoscopic resection and radiotherapy techniques, HN cancer patients with early esophageal neoplasms can be managed without surgical interventions to allow for a better quality of life. However, there are currently no standardized surveillance protocols with regards to the interval and therapeutic options for primary HN cancers and esophageal SPTs. In terms of personalized medicine, the treatment strategy should be individualized and discussed by a multidisciplinary team involving gastroenterologists, endoscopists, oncologists, radiologists, and HN and chest surgeons. Most of the enrolled studies in this review were retrospective or case-control design and the results might be influenced by the bias upon independent literature review. More well-designed prospective studies are warranted to establish the most appropriate treatment and surveillance programs to improve overall outcomes for HN cancer patients with esophageal SPTs.

## Author Contributions

Conceptualization, C-SC, L-JL and P-WS; Methodology, C-SC, L-JL and P-WS; Software, C-SC and L-JL; Validation, C-SC, L-JL, C-YW, W-CL, C-HH, T-HL, C-YL and P-WS; Formal analysis, C-SC and L-JL; Investigation, C-SC, L-JL, C-YW, W-CL, C-HH, T-HL, C-YL and P-WS; Resources, C-SC, L-JL, C-YW, W-CL, C-HH, T-HL, C-YL and P-WS; Data curation, C-SC, L-JL and P-WS; Writing—original draft preparation, C-SC; Writing—review and editing, C-SC, L-JL, D-YK and P-WS; Visualization, C-SC, L-JL, C-YW, W-CL, C-HH, T-HL, C-YL and P-WS; Supervision, C-SC, L-JL and P-WS; Project administration, C-SC; Funding acquisition, C-SC and P-WS. All authors have read and agreed to the published version of the manuscript.

## Conflict of Interest

The authors declare that the research was conducted in the absence of any commercial or financial relationships that could be construed as a potential conflict of interest.

## Publisher’s Note

All claims expressed in this article are solely those of the authors and do not necessarily represent those of their affiliated organizations, or those of the publisher, the editors and the reviewers. Any product that may be evaluated in this article, or claim that may be made by its manufacturer, is not guaranteed or endorsed by the publisher.
